# Carpological Analysis of Two Endemic Italian Species: *Pimpinella anisoides* and *Pimpinella gussonei* (Apiaceae)

**DOI:** 10.3390/plants12051083

**Published:** 2023-03-01

**Authors:** Valentina Lucia Astrid Laface, Carmelo Maria Musarella, Gianmarco Tavilla, Salvatore Cambria, Gina Maruca, Gianpietro Giusso del Galdo, Giovanni Spampinato

**Affiliations:** 1 Department of AGRARIA, Mediterranean University of Reggio Calabria, 89122 Reggio Calabria, Italy; 2 Department of Biological, Geological and Environmental Sciences, University of Catania, Via A. Longo 19, 95125 Catania, Italy; 3 Institute of Biosciences and Bioresources, National Research Council, Via G. Amendola 165/a, 70126 Bari, Italy

**Keywords:** endemism, fruit, morphometry, Sicily, Southern Italy, Umbelliferae

## Abstract

This study aims to clarify the taxonomic doubts, which have varied over the centuries, on the only two endemic species of the genus *Pimpinella* growing in Italy: *P. anisoides* and *P. gussonei*. For this purpose, the main carpological characters of the two species were examined, analyzing the external morphological traits and their cross-sections. Fourteen morphological traits were identified, obtaining datasets for the two groups using 40 mericarps (20 per species). The obtained measurements were subjected to statistical analysis (MANOVA and PCA). Our results highlight that at least 10 of the 14 morphological traits analyzed support the distinction between *P. anisoides* and *P. gussonei*. In particular, the following carpological characters are very significant ways to distinguish between the two species: monocarp width and length (Mw, Ml), monocarp length from base to maximum width (Mm), stylopodium width and length (Sw, Sl), length/width ratio (l/w) and cross-section area (CSa). In particular, the fruit of *P. anisoides* is larger (Mw 1.61 ± 0.10 mm) than that of *P. gussonei* (Mw 1.27 ± 0.13 mm), the mericarps of the first species are longer (Ml 3.14 ± 0.32 vs. 2.26 ± 0.18 mm) and the cross-section area (CSa) of *P. gussonei* is larger (0.92 ± 0.19 mm) than that of *P. anisoides* (0.69 ± 0.12 mm). The results also highlight the importance of the morphological traits of the carpological structures for the specific discrimination of similar species. The findings of this study contribute to an evaluation of the taxonomic significance of this species within the genus *Pimpinella*, and also provide valuable information for the conservation of these two endemic species.

## 1. Introduction

The genus *Pimpinella* L. is one of the most complex and species-rich of the family Apiaceae Lindl. Despite studies by numerous authors, its taxonomy is still not fully resolved [1,2,3,4,5]. Fernández Prieto et al. [6], in their study of the *Pimpinella* species from Western Europe, pointed out that it is a polyphyletic group. The genus includes about 150–180 taxa distributed in temperate and subtropical regions of Eurasia and Africa, South America, and western North America [6,7,8,9].

In the taxonomic studies on this genus, various morphological characters were considered [5,7,9,10], focusing on the micromorphological and anatomical characteristics of the fruit [11,12,13,14,15,16,17,18]. Carpological characters have traditionally been used for a long time in the systematics of Apiaceae, being among the most accessible to researchers [1,3]. The fruit anatomy and morphology have considerable taxonomic and evolutionary importance and, together with the molecular phylogenetic analyses, allow defining a stable taxonomy and an evolutionary history of species and genera [17]. Molecular phylogenetic analyses have raised new questions and, in many cases, have initiated a review of generic boundaries [5,6]. Kljuykov et al. [18] highlighted the requirements of morphological analyses for the characterization of taxa, re-evaluating the value of single character states and refining the terminology.

*Pimpinella* fruit is a bilocular schizocarp, consisting of two (homomorphic) mericarps, each with five ribs, dorsally compressed (hemispherical), flanked in the ventral part and joined with the carpophore bifid to the middle, which usually splits at maturity [3,4,19]. The fruit is rich in various essential oils, making these species aromatically valuable [20].

In Italy, Bartolucci et al. [21] reported seven native species and Galasso et al. [22] one casual alien; subsequent updates recognize 10 taxa, of which 9 are native (including 3 subspecies) and 1 casual alien [23]. Stinca and Ricciardi [24,25], on the other hand, recognise 12 Italian taxa in the genus *Pimpinella*, including 11 native species and subspecies and 1 alien. Among the taxa occurring in Italy, only two species are endemic, namely *Pimpinella anisoides* V.Brig. and *Pimpinella gussonei* (C.Presl) Bertol.

*Pimpinella anisoides*, described by Vincenzo Briganti in 1802 [26], grows in Southern Italy (Campania, Basilicata, Latium, Apulia, Calabria, and Sicily) [21,23,24,25] in the mesophylous woodland belts of *Quercus* spp. and *Castanea sativa* Mill. In Calabria, this species has an important economic value in the niche goods market: in fact, it is used to flavor several bakery products and some typical liqueurs [26,27,28], while in Basilicata, the fruits called “ciminielli” are used for the preparation of traditional sweets [29].

**Figure 1:**
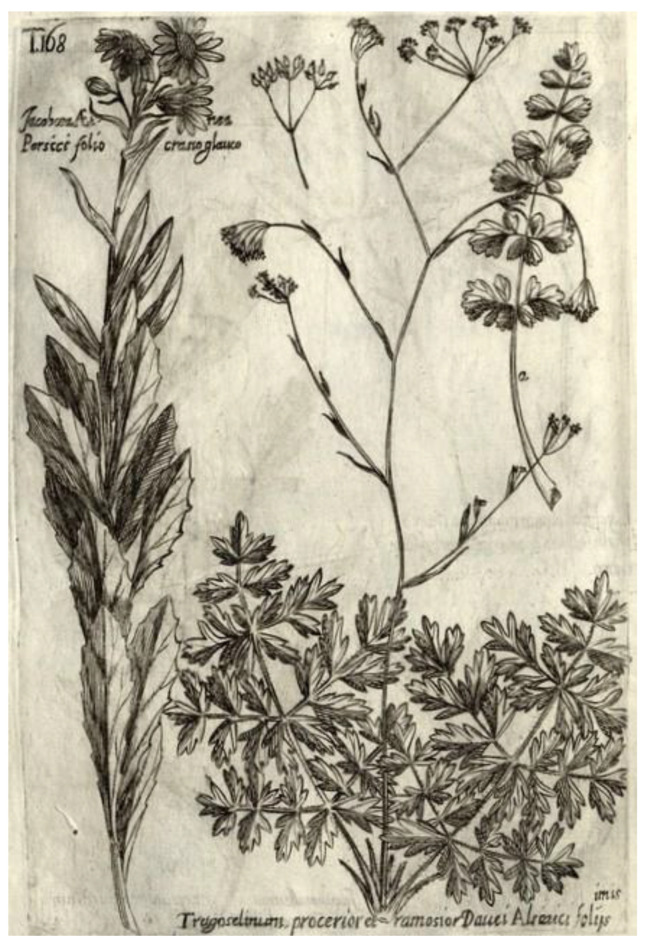
Iconography of Cupani’s *Panphyton Siculum* of 1713 [35], attributed to *Tragium gussonei* (on the right) by Presl [36], Gussone [37], Bertoloni [39].

*Pimpinella gussonei* is a species exclusive to Sicily [21,23,24,25], an island that is well-known as a hotspot for endemic species [30,31,32]. The species grows from the sea level up to 1500 m asl in uncultivated farmlands, clearings and coppices [33]. The taxonomic history of this species is rather controversial. The earliest information can be found in Cupani [34], who in his pre-Linnean work *Hortus Catholicus* reports the species in Enna (Sicily, Italy) as “*Pimpinella hircina, faxifraga*”, and later [35] in “*Panphyton Siculum*” reports a detailed iconography and describes the species as “*Tragoselinum procerior et ramosior, Dauci alsatici foliis imis*”, i.e., as “a taller, ramose tragoselinum with basal leaves of *Daucus alsaziano*” (Figure 1). Several authors of Sicilian or Italian floras quote Cupani as Presl [36] who describe the species in the Linnean system, Gussone [37,38] in “*Plantae rariores*” and Bertoloni in “*Flora italica*” [39].

Presl, in “*Deliciae Pragenses, Historiam Naturalem Spegtantes*”, described the species as *Tragium gussonii* [36], dedicating it to Giovanni Gussone. Subsequently, Gussone [37] reported it in Calabria, near Reggio, highlighting how this species is strongly akin to *Pimpinella anisoides* as described by Briganti, so much so that it misled him on the identification of some specimens sent to him by foreign botanists. He also highlights the differential characteristics of the two species: “*…T. Gussonii, caulis semper dichotomus; umbellae radii 4–5; fructus griseo-villosi; styli divaricato-reflexi, stigmatibus capitatis: dum in P. anisoide, caulis dichotomus vel ramis aliquando ternis (semiverticillatis); umbellae radii 5–7*; *fructus glaberrimi, virentes; styli erecto-patentes, stigmatibus vix incrassatis*”. That is: “*T. Gussonii*, stem always dichotomous; umbels with 4–5 rays; fruit grey-haired; styles divaricate-reflexed, with capitate stigmas: whereas in *P. anisoide* the stem is dichotomous or the branches are sometimes triple (semi-verticillate); umbels with 5–7 rays; fruits are very glabrous, green; styles erect-patent, the stigmas barely enlarged”.

Bertoloni [39] considers *Tragium gussonei* a synonym of *Pimpinella gussonii*, and reports the species being in the Sicilian localities of Palermo, San Martino, Carini, Ficuzza and Alcamo, as already mentioned by Gussone [38], who also reported the species in Castellammare, Mazzara, Marsala, Termini, Polizzi, Caltavuturo, Castrogiovanni, Catania, Nicolisi and Randazzo.

Caruel [40] considers both *P. gussonei* and *P. anisoides* to be synonyms of *Apium anisoides*. Fiori [41] considers *P. gussonei* to be a variety of *P. anisoides*, pointing out that the distinctive characters of the varieties are attributable to carpological differences.

Tutin [42] does not recognise *P. gussonei* either as a species or as a subspecies or variety, believing that the carpological character of the pubescence of the fruit is part of the variability of *P. anisoides*. Pignatti [29] and Stinca and Ricciardi [24,25] include *P. gussonii* Bertol. in the variability of *P. anisoides*, pointing out that the taxonomic value of those populations with fruits provided with appressed hairs should be further investigated. Conti et al. [43] only recognized *P. anisoides*. Peruzzi et al. [44] distinguish the two species and according to Art. 60.7 of ICN specify that the spelling of the specific epithet originally published as “*gussonii*” is to be corrected to “*gussonei*”. Bartolucci et al. [21] and their updates [23] consider *P. anisoides* V.Brig. and *P. gussonei* (C.Presl) Bertol. as two distinct species. Govaerts et al. [45], POWO [46] and Fernández Prieto et al. [6] consider *P. gussonei* (C.Presl) Bertol. a synonym of *P. anisoides* V.Brig. Furthermore, again according to Govaerts et al. [45] and POWO [46], *Tragium gussonei* C.Presl is a synonym of *Pimpinella villosa* Schousb., a species with a range including Morocco, the Azores Islands, Portugal and Spain. Presl [36] himself, in describing the species, emphasizes the profound affinity with *P. villosa* while highlighting its differences “*Affinis forte T. villoso (Pimpinellae villosae Schousb.) sed differre videtur: pinnulis ovatis profunde dentatis, petalis glabris…*” that is “… strongly akin to *T. villoso* (*Pimpinellae villosae* Schousb.), but appears to differ: deeply toothed ovate fins, glabrous petals…”. Differences also highlighted by Gussone, who considers *P. gussonei* (sub *Tragium gussonii*) glabrous throughout [37]. Moreover, several authors, including Presl [36], point out that *Pimpinella gussonei* also has numerous affinities with *Pimpinella bubonoides* DC. Gussone [47] considers *P. bubonoides* as a synonym of *Pimpinella anisoides*, while Gussone [37] again highlights the distinguishing characteristics of *Pimpinella gussonei* (sub. *T. gussonii*) with respect to *P. bubonoides*, indicating that the species does not have large basal leaves, nor alternate and composite cauline leaves. Bertoloni [39] considers *P. bubonoides* as a synonym of *P. gussonii*.

The historical excursus shows that the taxonomic events related to *Pimpinella gussonei* and *Pimpinella anisoides* are very complex and still unresolved.

This study provides a first characterization and differentiation between these two endemic species with the analysis of the carpological traits. This distinction is also helpful for economic purposes to identify commonly marketed fruits and counteract adulteration

## 2. Results and Discussion

### 2.1. Carpological Analysis

In agreement with Cano et al. [48], fruit morphometry is a good tool for distinguishing similar species that differ in a few characters that are not always unique.

In the case of *P. anisoides* and *P. gussonei*, the schizocarp of both species is similar, oblong-ovoid in shape, with five grooves between five prominent, and more or less prominent dorsal ribs; it consists of two homomorphic mericarps, having a flat ventral surface, and is joined by a carpophore bifid to the middle, usually splitting when ripe [19].

**Figure 2:**
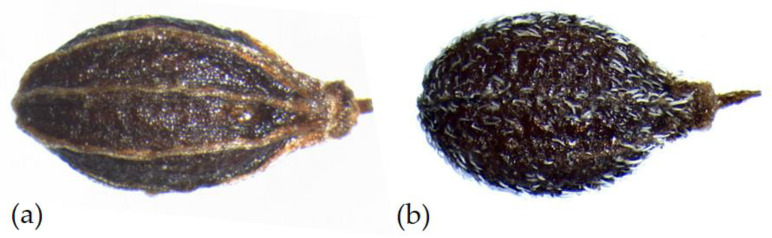
Mericarps of: (**a**) *Pimpinella anisoides* V.Brig. and (**b**) *Pimpinella gussonei* (C.Presl) Bertol.

In *P. anisoides*, the fruit is glabrous throughout, dark brown–black in color, having an average length of 3.14 (±0.59) mm and a width of 1.61 (±0.16) mm; the average length/width ratio is 1.94, and the distance between the base and the maximum width is 1.25 (±0.20) mm. The stylopodium has a length of 0.38 (±0.13) mm and a width of 0.47 (±0.14) mm. In *P. gussonei*, the fruit is pubescent due to numerous single hairs appressed to the dorsal part, the ventral part is glabrous or with a few scattered hairs and grey–black in color; on average it is 2.26 (±0.33) mm long and 1.27 (±0.27) mm wide, the average length/width ratio is 1.70, and the distance between the base and the maximum width is 0.91 (±0.19) mm. The stylopodium has a length of 0.30 (±0.07) mm and a width of 0.25 (±0.42) mm (Figure 2; Table 1).

The cross-section of the mericarps in both species is similar and shows morphological characteristics according to Laface et al. [20]; the dimensions of the different characters examined differ.

**Figure 3:**
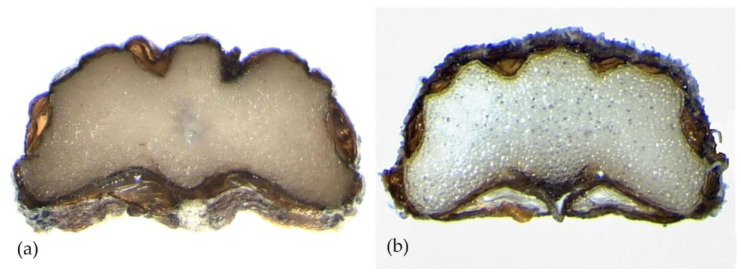
Mericarps cross-section of: (**a**) *Pimpinella anisoides* V.Brig. and (**b**) *Pimpinella gussonei* (C.Presl) Bertol.

In *P. anisoides*, the cross-section area is 0.69 mm^2^, with a thickness 0.74 (±0.17) mm long and 1.50 (±0.33) mm wide, and the average width/thickness ratio is 2.05. The commissural vittae are 0.53 (±0.18) mm long and 0.17 (±0.04) mm wide with a width/thickness ratio of 3.25. The commissure width is 1.18 (±0.31) mm. In *P. gussonei*, the cross-section area is 0.92 (±0.33) mm^2^, the thickness is 0.85 (±0.21) mm long and 1.64 (±0.41) mm wide, and the average width/thickness ratio is 1.94. The commissural vittae are 0.55 (±0.12) mm long and 0.15 (±0.05) mm thick with a width/thickness ratio of 3.73. The commissure width is 1.23 (±0.28) mm (Figure 3, Table 1).

### 2.2. Statistical Analysis

**Figure 4:**
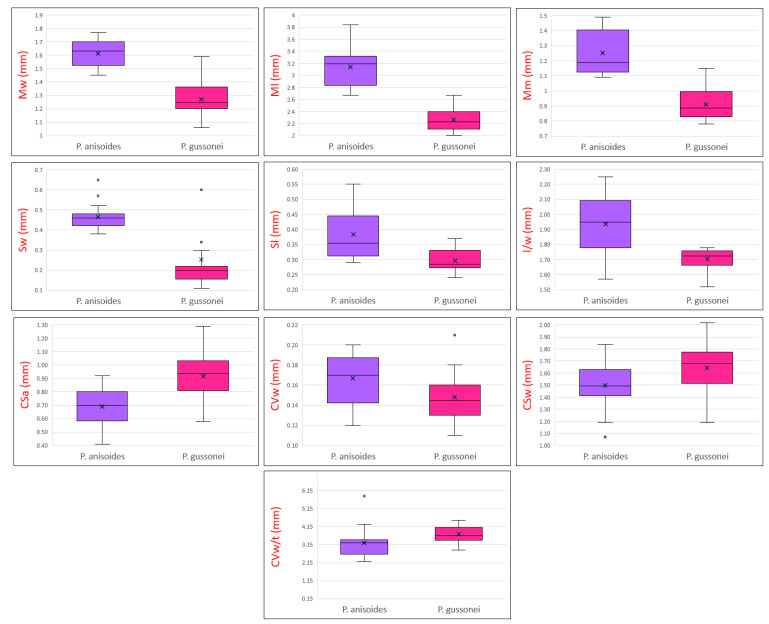
Boxplots of statistically significant fruit morphological characters of *Pimpinella anisoides* V.Brig. and *Pimpinella gussonei* (C.Presl) Bertol. Character abbreviations: fruit width (Mw); fruit length (Ml); length from the lower end to the point of maximum width (Mm); stylopodium width and length (Sw, Sl); length/width ratio (l/w); area per section (CSa); commissural vittae width (CVw); section width (CSw); commissural vittae width/thickness ratio (CVw/t). The terminology of fruit parts and section is in accordance with Kljuykov et al. [19], Akalın et al. [9], Yeşil et al. [18].

The results of the multivariate analysis of the characters are summarized in Table 1 and shown in the boxplots in Figure 4. The MANOVA multivariate analysis used to define the significance of the analyzed characters, shows that Mw, Ml, Mm, Sw, Sl, l/w and CSa, are highly significant (*p* < 0.001), while CSw, CVw and CV w/t are not very significant (*p* < 0.05), and the characters CVt, CCSl and CS w/t are not significant. The F values also show that the two examined species display different morphological traits, some of which are more evident: Mw (F = 90.902), Ml (F = 112.899), Mm (F = 76.237), Sw (F = 21.360), Sl (F = 18.878), l/w (27.166) and CSa (F = 17.867) are more differentiated and CSw (F = 5.154), CVw (F = 5.636) and CV w/t (F = 5.086) are not very differentiated. The characters found to be non-significant, CSt, CVt, CCSl and CS w/t, have values of F < 1. The boxplots (Figure 4) allow us to visualize the centre and distribution of the data, and highlight some outliers for the characters Sw, CVw and CV w/t.

**Figure 5:**
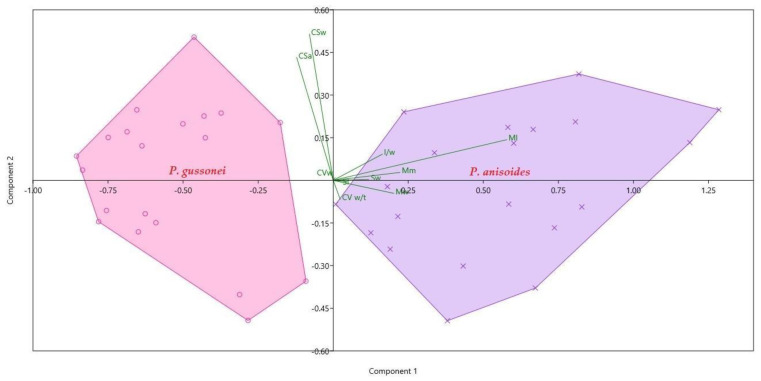
PCA based on 10 continuous quantitative traits’ significant results for *Pimpinella anisoides* V.Brig. and *Pimpinella gussonei* (C.Presl) Bertol. fruits (see Table 1). The relative contribution of each variable is shown in green.

The PCA (Figure 5) clearly separates the fruits of *P. gussonei* and *P. anisoides*, showing a strong distinction between the carpological characters of the two species. In addition, the PCA shows that the first five characters with highest significance (Mw 76% variance, Ml 12% variance, Mm 4% variance, Sw 4% variance), explain 96% of the total variance. The biplot (Figure 5) also shows that some characters (Ml, Mm, Sw, Mw, l/w) contribute better to the characterization of *P. anisoides* than *P. gussonei*. On the whole, the characters concerning the external morphology of the fruit allow better distinction between the two species than the characters concerning the section of the fruit.

The obtained results highlight that the two species have similar carpological characteristics but have different size characters (Figure 4). The fruit of *P. anisoides* is larger (Mw 1.61 ± 0.10 mm) than that of *P. gussonei* (Mw 1.27 ± 0.13 mm); in addition, the mericarps of the first species are longer (Ml 3.14 ± 0.32 vs. 2.26 ± 0.18 mm). The stylopodium of *P. anisoides* is wider (Sw 0.47 ± 0.06 mm) than that of *P. gussonei* (0.25 ± 0.19 mm). The cross-section area (CSa) of *P. gussonei* is larger (0.92 ± 0.19 mm) than that of *P. anisoides* (0.69 ± 0.12 mm). The commissural vittae width/thickness ratio (CV w/t) is 3.25 ± 0.84 in *P. anisoides* and 2.73 ± 0.45 in *P. gussonei,* showing a more compressed shape in the mericarp of the first species. Overall, the mericarps of *P. anisoides* appear slightly more oblong (l/w-length/width ratio = 1.94) than those of *P. gussonei* (l/w-length/width ratio = 1.70). In agreement with Gussone [37], all the samples of *P. gussonei* fruit are grey tomentose, whereas those of *P. anisoides* are glabrous throughout.

In addition, the carpological characters of *P. anisoides* and *P. gussonei* were compared with those of other species of the genus *Pimpinella* growing in Italy (Table 2).

The comparison in Table 2 shows that *P. gussonei* and *P. anisoides* are the only two natives to be of aromatic interest, apart from *P. anisum* of the species introduced for cultivation. Both species have oblong-ovoid fruits, similar to those of *P. saxifraga*, but this is not aromatic. The fruit size of *P. anisoides* is similar to that of *P. major*, which, however, has ovoid fruits. *P. gussonei* is similar in size to *P. peregrina*, which, however, has elliptic and hispid fruits.

Traditionally, the *Pimpinella* genus differs from related genera of the Apiaceae family due to a series of characteristics that are mainly of carpological type. The fruits, no longer than 5 mm, are slightly longer than wide, ovoid-oblong to subglobose, laterally compressed, with inconspicuous filiform primary ridges [29,42]. The phylogenetic analysis conducted by Fernández Prieto et al. [6] shows that in the *Pimpinella* genus, it is not possible to identify sections as proposed by de Candolle [49] and Wolff [1]. On the other hand, Fernández Prieto et al. [6] separate some species formerly included in the *Pimpinella* genus, attributing them to related genera: *Spiroceratium* H.Wolff and *Parapimpinella* Fern. Prieto et al.

## 3. Materials and Methods

### 3.1. Fruit Collection and Morphometric Data

**Figure 6:**
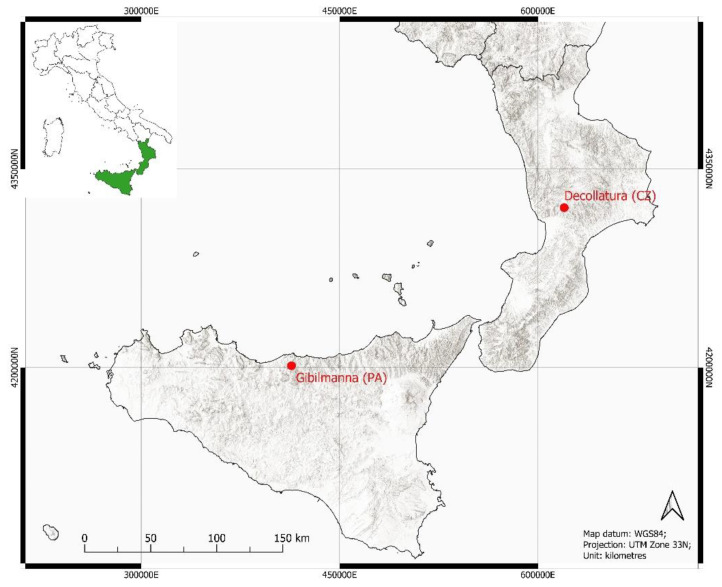
Map localities where fruit collections were carried out: Decollatura (Catanzaro—CZ) in Calabria for *Pimpinella anisoides* V.Brig. and Gibilmanna (Palermo—PA) in Sicily for *Pimpinella gussonei* (C.Presl) Bertol.

The analysed fruits were collected from different localities: *P. anisoides* in Calabria in the Presila Catanzarese near Decollatura (CZ) and *P. gussonei* in Sicily near Gibilmanna (PA) (Figure 6).

The samples were collected in August 2022 during the period of maximum fruit filling, the collection was randomized within the sampled population for both species. At the same time, herbarium samples of the two species were collected and stored at the herbarium of the Mediterranean University of Reggio Calabria (REGGIO) for those of *P. anisoides* and at the herbarium of the University of Catania (CAT) for those of *P. gussonei* (acronyms follow Thiers [50]). The two species are very similar, in fact they are scapose hemicryptophytes, with enlarged woody root, erect stems, up to 80 cm tall, tripinnatifid leaves with lanceolate final segments, usually provided with 1–2 teeth, umbel-shaped inflorescence without involucre and involucel with 5–6 rays, flowers with white petals. The fruits were measured, in all their parts, by means of a stereomicroscope equipped with an Invenio 5SII HD camera connected to a computer where, by means of the DeltaPix inSight© software, it was possible to reconstruct the image using a multifocal system, and to determine the character measurements of the fruit. Morphometric analyses were carried out on a sample of 20 fruits and 20 cross-sections. The fruits measured were those that were found on a first macroscopic analysis to be perfectly ripe, well-formed and free of defects, caused by mechanical damage or insects. The following were measured in detail: fruit width (Mw), fruit length (Ml), length from the lower end to the point of maximum width (Mm), stylopodium width and length (Sw, Sl) and the length/width ratio (l/w). Fruit cross-sections were also observed to define their micromorphology, and the following were measured in detail: section width and thickness (CSw, CSt) and their ratio (CSw/t), commissural vittae width and thickness (CVw, CVt) and their ratio (CVw/t), commissure width (CCSl). The area per section (CSa) was also measured. The terminology of fruit parts and section is in accordance with Kljuykov et al. [19], Akalın et al. [9], Yeşil et al. [18]. Information of the carpological characters of the other species of the *Pimpinella* genus occurring in Italy is taken from Pignatti et al. 2018, Yeşil et al. [18] and Tutin et al. [42].

### 3.2. Statistical Analysis

The data from the measurements were collected and analysed in a matrix in Excel©2019, the mean and standard deviation of the 14 characters examined were also calculated. To define the significance of the data, statistical differences were obtained by MANOVA multivariate analysis of the 14 characters (dependent variable) measured in both species (independent variable). SPSS^®^ 23.0 software (SPSS Inc., Chicago, IL, USA) was used for this purpose. Quantitative raw data were subjected to Principal Component Analysis (PCA) with a covariance matrix based on morphological features found significant by the multivariate MANOVA analysis. The statistical significance of the Principal Components (PCs) was analysed by the Broken Stick method, using Past 4.3^®^ software. [51,52]. Boxplots were carried out with Excel© 2023 using only those characters found significant by the multivariate MANOVA analysis. In each boxplot, the centre and distribution of the data are highlighted.

## 4. Conclusions

This study contributes to the initial characterization of and differentiation between *P. anisoides* and *P. gussonei*, two often confused species that locally have a certain economic importance for the fruits used to flavor foods and drinks. The carpological analysis of the two species endemic to Italy revealed differences in size and morphological and anatomical fruit characteristics. Overall, the results allow a simple distinction of the two species in agreement with Presl [36], Gussone [37,38] and Bertoloni [39], and highlight the importance of carpological characters for the taxonomy of the *Pimpinella* genus. However, in order to determine the relationship between the two endemic Italian *Pimpinella* species more precisely, extensive studies are still needed, including the analysis of the morphological characteristics of the vegetative and reproductive organisms as well as DNA sequences.

## Figures and Tables

**Table 1:** Comparison of the monocarp characters of *Pimpinella anisoides* V.Brig. and *Pimpinella gussonei* (C.Presl) Bertol. used in morphometric analyses. Quantitative numerical values (mm) are expressed as mean ± SD. Significant data (*p*) are indicated in bold. Fisher’s F highlights the ratio of the variance between the two samples.

Code	Description	*P. anisoides*	*P. gussonei*		
		Mean ± SD	Mean ± SD	F	*p* (Statistical Significance)
**Mw**	**Monocarp width (mm)**	**1.61 ± 0.10**	**1.27 ± 0.13**	**90.902**	***
**Ml**	**Monocarp length (mm)**	**3.14 ± 0.32**	**2.26 ± 0.18**	**112.899**	***
**Mm**	**Monocarp length from base** **to maximum width (mm)**	**1.25 ± 0.14**	**0.91 ± 0.10**	**76.237**	***
**Sw**	**Stylopodium width (mm)**	**0.47 ± 0.06**	**0.25 ± 0.19**	**21.356**	***
**Sl**	**Stylopodium length (mm)**	**0.38 ± 0.08**	**0.30 ± 0.04**	**18.878**	***
**l/w**	**Length/width ratio**	**1.94 ± 0.19**	**1.70 ± 0.07**	**27.166**	***
**CSw**	**Width of cross-section (mm)**	**1.50 ± 0.17**	**1.64 ± 0.21**	**5.154**	*
CSt	Thickness of cross-section (mm)	0.74 ± 0.09	0.85 ± 0.12	2.857	n.s.
**CSa**	**Cross-section area (mm^2^)**	**0.69 ± 0.12**	**0.92 ± 0.19**	**17.867**	***
**CVw**	**Width of commissural vittae (mm)**	**0.17 ± 0.03**	**0.15 ± 0.02**	**5.636**	*
CVt	Thickness of commissural vittae (mm)	0.53 ± 0.10	0.55 ± 0.06	0.389	n.s.
CCSl	Commissure width (mm)	1.18 ± 0.17	1.23 ± 0.13	0.937	n.s.
CS w/t	Cross-section width/thickness ratio	2.05 ± 0.31	1.94 ± 0.23	1.651	n.s.
**CV w/t**	**Commissural vittae width/thickness ratio**	**3.25 ± 0.84**	**2.73 ± 0.45**	**5.086**	*

* = *p* < 0.05; *** = *p* < 0.001; n.s. = *p* > 0.05.

**Table 2:** Comparison of the monocarp characters of *Pimpinella* species present in Italy.

Taxon	Fruit Length (mm)	Indumentum	Shape of Fruit	Aromatic
*Pimpinella anisoides* V.Brig.	2.5–3.7	glabrous	oblong-ovoid	yes
*Pimpinella anisum* L.	3.9–4	strigose	ovoid	yes
*Pimpinella gussonei* (C.Presl) Bertol.	1.9–2.6	pubescens	oblong-ovoid	yes
*Pimpinella lutea* Desf.	3.5–4	glabrous	eliptic	no
*Pimpinella major* (L.) Huds.	2.5–3.5	glabrous	ovoid	no
*Pimpinella peregrina* L.	1.9–2	hispid	eliptic	no
*Pimpinella saxifraga* L.	2.1–2.2	glabrous	oblong-ovoid	no
*Pimpinella tragium* Vill.	2	strigose	ovoid	no

## Data Availability

The data are contained within the article.
